# Comprehensive analysis of KCTD family genes associated with hypoxic microenvironment and immune infiltration in lung adenocarcinoma

**DOI:** 10.1038/s41598-022-14250-6

**Published:** 2022-06-15

**Authors:** Yuan-Xiang Shi, Wei-Dong Zhang, Peng-Hui Dai, Jun Deng, Li-Hong Tan

**Affiliations:** 1 Institute of Clinical Medicine, Hunan Provincial People’s Hospital, The First Affiliated Hospital of Hunan Normal University, Changsha, 410005 Hunan People’s Republic of China; 2 Respiratory Medicine, Hunan Provincial People’s Hospital, The First Affiliated Hospital of Hunan Normal University, Changsha, 410005 Hunan People’s Republic of China; 3 Department of Pathology, Hunan Provincial People’s Hospital, The First Affiliated Hospital of Hunan Normal University, Changsha, 410005 Hunan People’s Republic of China; 4 Department of Pharmacy, Hunan Provincial People’s Hospital, The First Affiliated Hospital of Hunan Normal University, Changsha, Hunan 410005 People’s Republic of China

**Keywords:** Biomarkers, Oncology

## Abstract

To obtain novel insights into the tumor biology and therapeutic targets of LUAD, we performed a comprehensive analysis of the KCTD family genes. The expression patterns and clinical significance of the KCTD family were identified through multiple bioinformatics mining. Moreover, the molecular functions and potential mechanisms of differentially expressed KCTDs were evaluated using TIMER 2.0, cBioPortal, GeneMANIA, LinkedOmics and GSEA. The results indicated that the mRNA and protein expression levels of KCTD9, KCTD10, KCTD12, KCTD15 and KCTD16 were significantly decreased in LUAD, while those of KCTD5 were significantly increased. High KCTD5 expression was significantly associated with advanced tumor stage, lymph node metastasis, TP53 mutation and poor prognosis. In addition, KCTD5 was positively correlated with CD8 + T cell, neutrophil, macrophage and dendritic cell infiltration. Additionally, KCTDs demonstrate promising prospects in the diagnosis of LUAD. Importantly, high KCTD5 expression was enriched in signaling pathways associated with the malignant progression of tumors, including the inflammatory response, the IL6/JAK/STAT3 signaling pathway, EMT and hypoxia. Further association analysis showed that KCTD5 was positively correlated with hypoxia-related genes such as HIF1. Overall, KCTDs can be used as molecular targets for the treatment of LUAD, as well as effective molecular biomarkers for diagnosis and prognosis prediction.

## Introduction

The incidence and mortality of lung cancer rank first among malignant tumors, and it has become an important disease endangering public health^1^. Lung adenocarcinoma (LUAD) is one of the most important subtypes. It is of great clinical significance to identify novel and reliable molecular targets for diagnosis and treatment of LUAD^2,3^. Ubiquitination has been widely studied in recent years, and we have also done some work in this field. The human family of potassium channel tetramerization domain-containing (KCTD) proteins contains 25 members, sharing a conserved BTB (Bric-a-brack, Tram-track, Broad complex) domain at the N-terminal^4^. Most KCTD proteins bind Cullin3-dependent E3 ubiquitin ligase via the BTB domain and are closely related to protein ubiquitination^5,6^. Ubiquitination is an important post-translational modification that regulates the localization, activity and stability of substrate proteins^6,7^. Previous studies have suggested that KCTD family genes are involved in the regulation of tumorigenesis^8^. However, the expression patterns, clinical applications, immune infiltration levels and genetic variations of KCTD family proteins have not been reported in LUAD.

In the current study, we first identified differentially expressed KCTDs (KCTD2, KCTD5, KCTD9, KCTD10, KCTD12, KCTD15, KCTD16 and KCTD21) in LUAD. Next, we investigated the correlation between the expression level of KCTDs and clinicopathological parameters. Furthermore, the clinical application of KCTDs in LUAD was discussed. Receiver operating characteristics (ROC) analysis and survival analysis showed that KCTDs had potential value in diagnosis and prognosis prediction. Importantly, the correlations between the expression of KCTDs and immune infiltration, as well as KCTDs genetic alterations, were analyzed in LUAD. Finally, gene set enrichment analysis (GSEA) and correlation analysis were used to preliminarily explore the molecular mechanisms of KCTDs regulating LUAD.

## Materials and methods

### Database and public platform

The Cancer Genome Atlas (TCGA) database integrates the gene expression and corresponding clinicopathological feature data of a variety of cancer patients (http://cancergenome.nih.gov)^9^. In this study, we extracted the mRNA expression profile (RNA-Seq, level 3) and clinicopathological data of lung cancer from TCGA database for ROC analysis and GSEA.

UALCAN is a convenient public tumor database (http://ualcan.path.uab.edu)^10^. In this study, the mRNA expression profile data of LUAD from the UALCAN and TCGA databases were used for differential expression analysis, including normal samples and LUAD samples with different tumor stages, lymph node metastasis statuses, and TP53 mutation statuses. We used TPM (transcripts per million) as the measure of expression. The LUAD protein expression profile provided by the Clinical Proteomic Tumor Analysis Consortium (CPTAC) dataset were used to confirm the protein expression of KCTD family genes^11^. Total protein expression levels of KCTD family genes were compared between LUAD samples and normal samples.

Gene Expression Profiling Interactive Analysis 2 (GEPIA2) is a website for the analysis and visualization of tumor and normal expression data (http://gepia2.cancer-pku.cn/)^12^. In this study, we used the GEPIA online tool to analyze the differential expression between LUAD and normal (match TCGA normal and GTEx data) samples and to perform correlation analysis of KCTD5 and hypoxia-related genes. The expression data were first transformed into log_2_ (TPM + 1) values for differential analysis. The parameters are as follows, differential methods: ANOVA, |log_2_FC| cutoff: 1, q-value cutoff: 0.01. The Pearson correlation coefficient was used to describe the degree of correlation between two genes. We also used GSCA (Gene Set Cancer Analysis) online database (http://bioinfo.life.hust.edu.cn/GSCA/#/) to evaluate the expression differences between tumor and normal samples of CENP family genes in LUAD^13^.

Kaplan–Meier Plotter was used to evaluate the effect of KCTD gene expression on overall survival (OS) in patients with LUAD (http://kmplot.com/analysis/)^14,15^. The patients were divided into a high expression group and a low expression group based on the median expression value. PrognoScan (http://dna00.bio.kyutech.ac.jp/PrognoScan/index.html) is a major survival analysis database with data mainly from Gene Expression Omnibus (GEO). We also used this database to assess the relationship between KCTDs expression and patient outcomes in lung cancer^16^.

TIMER 2.0 is a comprehensive database that systematically analyzes and visualizes the immune infiltration of multiple tumors (http://timer.cistrome.org/)^17^. In this study, TIMER 2.0 was used to evaluate the association between KCTD gene expression and immune infiltrates in LUAD. The Spearman correlation coefficient was used.

cBioPortal is a comprehensive platform for data mining, integration and visualization that was developed based on the TCGA database (http://www.cbioportal.org/)^18^. The genetic alterations of KCTD family members in LUAD were analyzed using cBioPortal. The parameters were set as follows: 1) select lung adenocarcinoma datasets "TCGA, Nature 2014", "TCGA, PanCancer Atlas" and "TCGA, Firehose Legacy"; 2) choose molecular profiles "Copy number alterations", "Structural variants" and "Mutations"; 3) enter gene list “KCTD2, KCTD5, KCTD9, KCTD10, KCTD12, KCTD15, KCTD16, KCTD21”. In this study, GeneMANIA was used to analyze the co-expression and interactions of KCTD family genes (http://www.genemania.org)^19^. Cytoscape software (v3.8.2) was used for the visualization^20^.

LinkedOmics is an online database that includes multi-omics data from all 32 TCGA cancer types and 10 CPTAC cancer cohorts (http://linkedomics.org/login.php)^21^. The co-expression of KCTD family genes was analyzed using RNAseq data from TCGA-LUAD database. Pearson correlation test was used to evaluate the correlation of co-expressed genes. In the “LinkFinder” module, the volcano plots and heat maps show genes that are positively/negatively associated with target genes.

### GSEA

GSEA v4.1.0 (http://www.gsea-msigdb.org/) was used to identify the possible mechanism by which KCTD5 regulates LUAD^22^. In this study, GSEA analysis was based on mRNA expression data of TCGA-LUAD. The Hallmark gene set file “h.all.v7.4.symbols.gmt” (MSigDB) was selected as the gene set database^22^. The number of permutations was set to 1000. Significance criteria were nominal *P*-value < 0.05 and false positive rate (FDR) < 0.05.

### ROC and statistical analysis

SPSS version19.0 was used for statistical analysis. Comparison of the mRNA expression and protein expression were performed using Student’s t-test (UALCAN), one-way ANOVA test (GEPIA2), Wilcoxon test (TIMER2.0), or Kruskal–Wallis test (LinkedOmics). Survival analysis was conducted through Kaplan–Meier Plotter with the log-rank test. Pearson test was used to assess the correlation between the expression of any two genes. The correlation between gene expression and immune infiltration was analyzed by Spearman's method. Logistic regression analysis and ROC analysis were applied to construct a tumor diagnostic model. The area under the curve (AUC) was calculated for each ROC curve. *P* < 0.05 was considered statistically significant. Besides, FDR < 0.05 was an additional criterion for GSEA.

## Results

### Expression pattern of KCTD family genes in patients with LUAD

**Figure 1:**
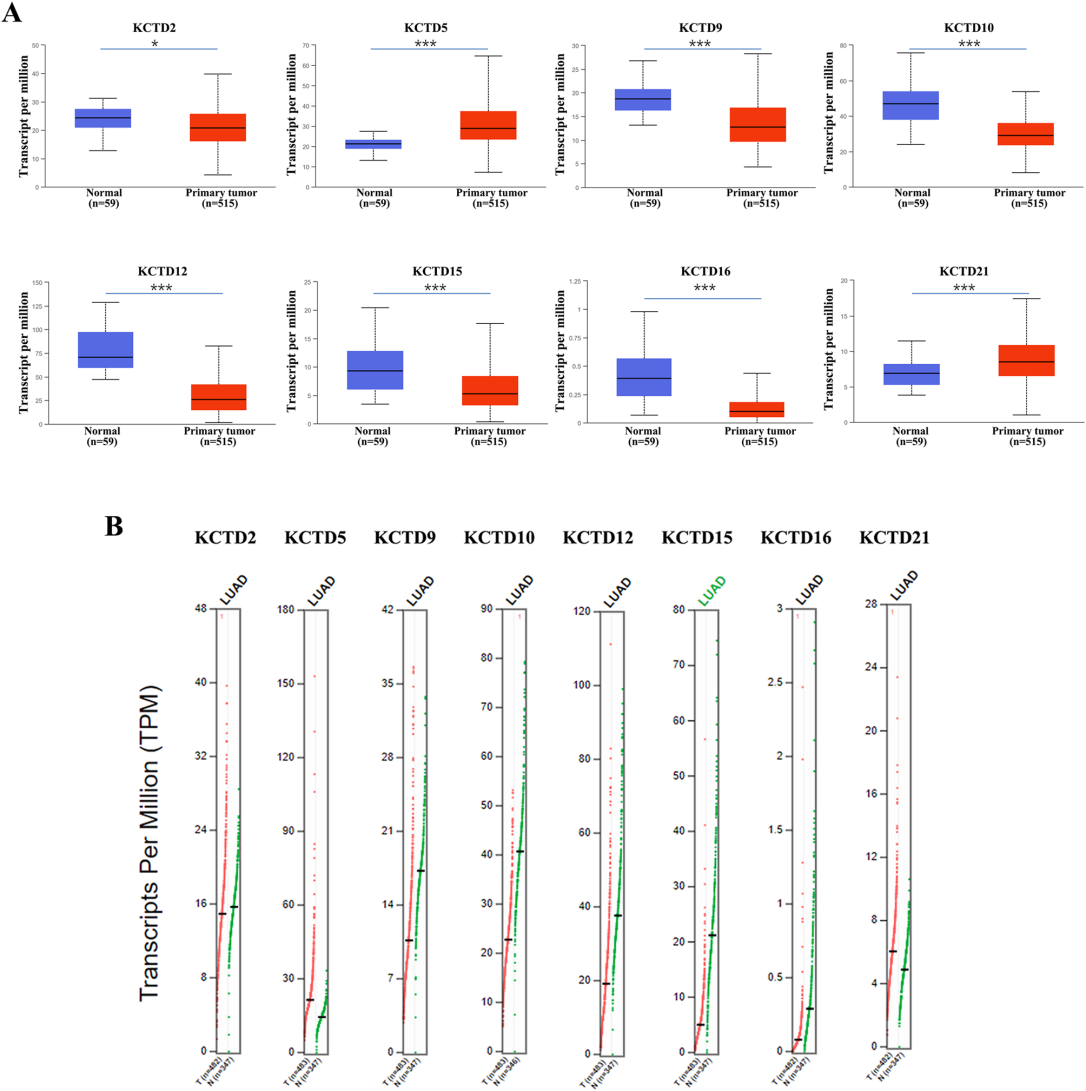
The mRNA expression of KCTD family genes in LUAD and normal lung tissues. (**A**) The mRNA expression of KCTD2, KCTD9, KCTD10, KCTD12, KCTD15 and KCTD16 in LUAD was significantly decreased, while KCTD5 and KCTD21 was significantly increased (UALCAN). (**B**) We verified the mRNA expression of KCTD family genes in LUAD using the GEPIA2 database. *P < 0.05, ***P < 0.001.

To identify the KCTD family genes that are differentially expressed in LUAD, differential expression analysis was performed (Student’s t-test). We extracted the mRNA expression profile data of LUAD from the TCGA database to detect the mRNA expression levels of KCTD family genes. Compared with normal tissues, the expression levels of KCTD2 (*P* < 0.05), KCTD9 (*P* < 0.001), KCTD10 (*P* < 0.001), KCTD12 (*P* < 0.001), KCTD15 (*P* < 0.001) and KCTD16 (*P* < 0.001) were significantly decreased in LUAD. In contrast, the expression levels of KCTD5 (*P* < 0.001) and KCTD21 (*P* < 0.001) were significantly higher in LUAD than in normal tissues (Fig. 1A).

Subsequently, we verified the mRNA expression of KCTD family genes in LUAD using the GEPIA2 database. The experimental results were consistent with those obtained from the TCGA database (Fig. 1B). The expression of KCTD family genes in LUAD was further confirmed in the GSCA database. KCTD5 and KCTD21 were significantly (FDR < 0.05) up-regulated in LUAD, while the expression levels of KCTD2, KCTD9, KCTD10, KCTD12, KCTD15 and KCTD16 were significantly (FDR < 0.05) lower than those in normal tissues (Supplementary Fig. 1).

**Figure 2:**
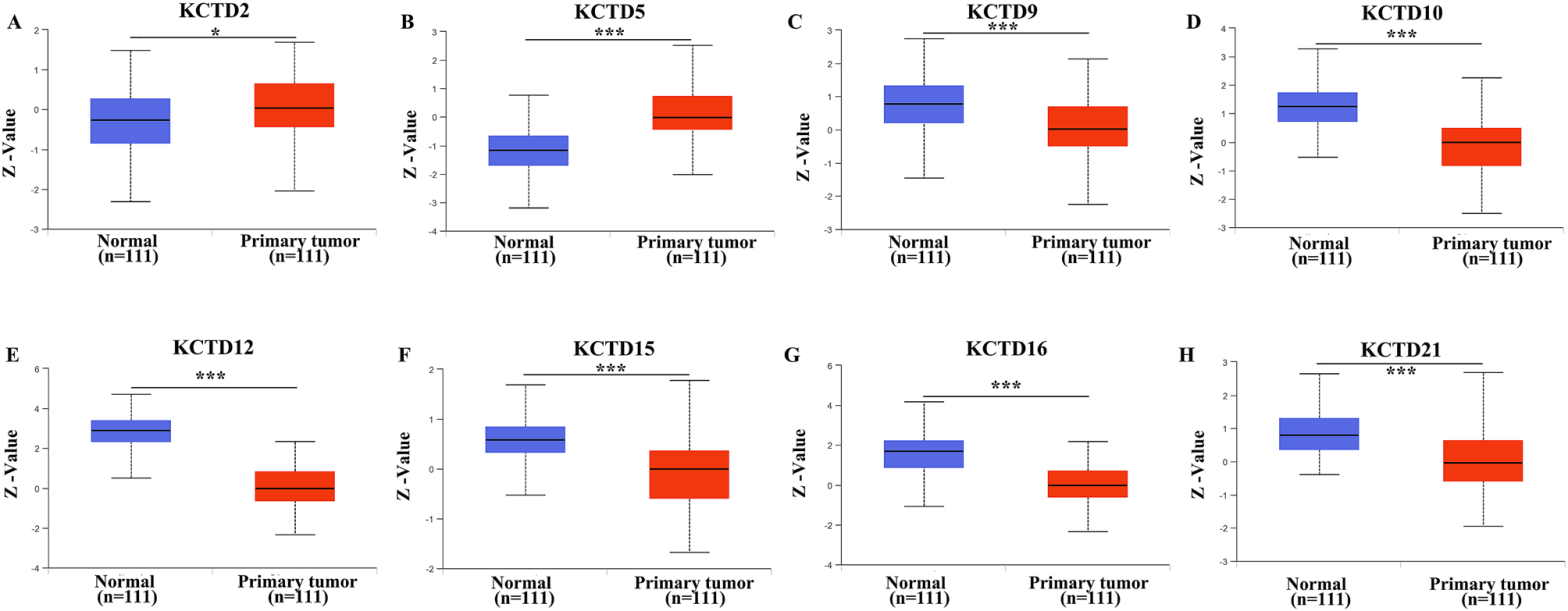
The protein expression of KCTD family genes in LUAD and normal lung tissues (UALCAN). KCTD9, KCTD10, KCTD12, KCTD15, KCTD16 and KCTD21 were low expressed in LUAD, while the expression level of KCTD2 and KCTD5 in LUAD were higher than that in normal tissues. *P < 0.05, ***P < 0.001.

Importantly, we further explored the protein expression of KCTD family genes in LUAD using the CPTAC database. The results suggested that KCTD9 (*P* < 0.001), KCTD10 (*P* < 0.001), KCTD12 (*P* < 0.001), KCTD15 (*P* < 0.001), KCTD16 (*P* < 0.001), and KCTD21 (*P* < 0.001) had low expression in LUAD, while the expression level of KCTD2 (*P* < 0.05) and KCTD5 (*P* < 0.001) in LUAD were higher than that in normal tissues (Fig. 2). We identified the expression patterns of KCTD family genes in LUAD at the mRNA level and the protein level. Surprisingly, we found that the expression of KCTD2 is inconsistent at the transcription level and translation level, the same situation also occurs on KCTD21.

### Correlation between the expression levels of KCTD family genes and the clinicopathological characteristics of LUAD patients

**Figure 3:**
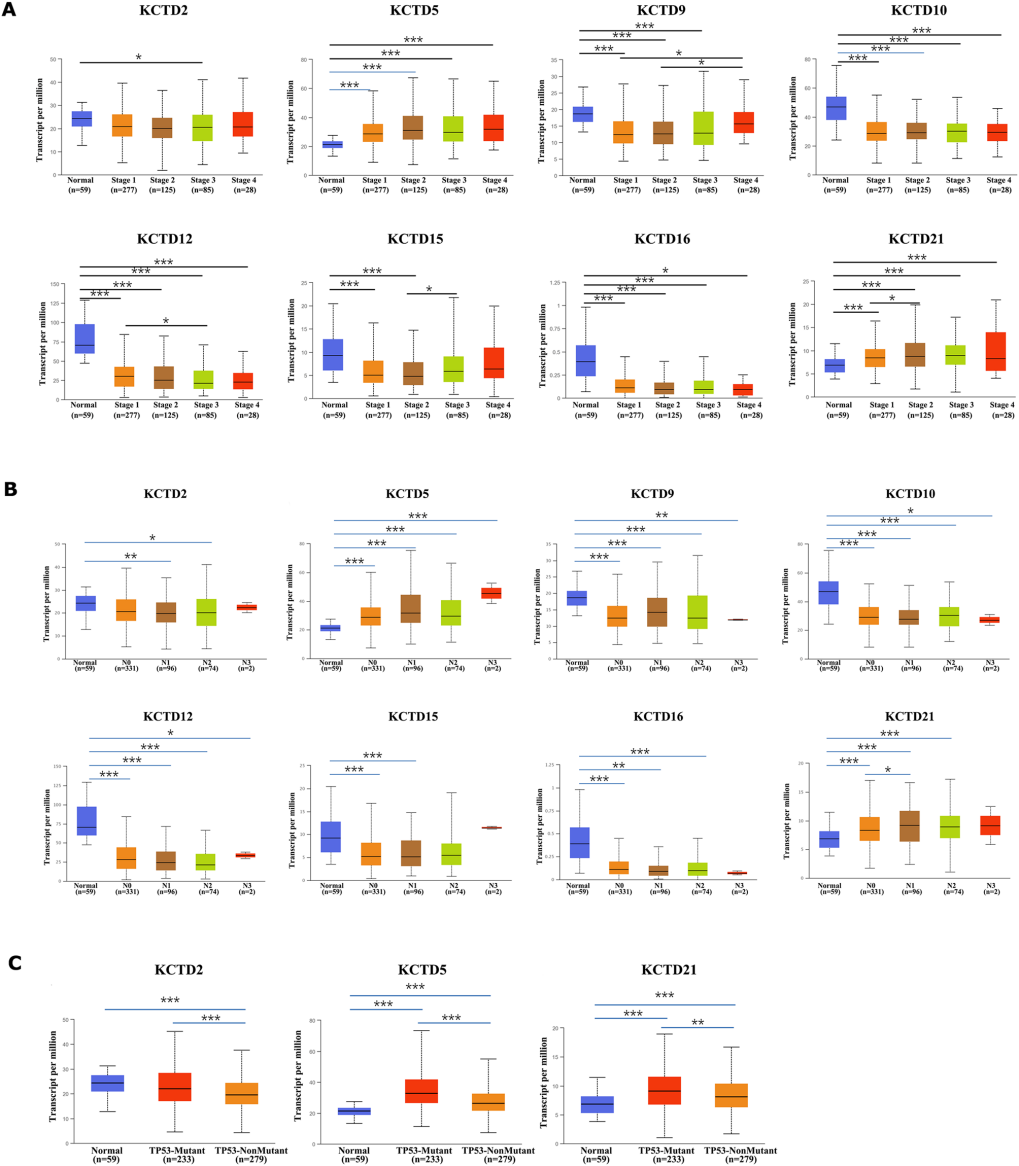
The correlation analysis between expression level of KCTD family genes and clinicopathological characteristics (UALCAN). (**A**) Tumor stage (stage 1, stage 2, stage 3 and stage 4). (**B**) Lymph node metastasis (N0, N1, N2 and N3). (**C**) TP53 mutation. *P < 0.05, **P < 0.01 and ***P < 0.001.

The correlation analysis between expression levels of KCTD family genes and clinicopathological characteristics (tumor stage, lymph node metastasis and TP53 mutation status) was performed (Student’s t-test). Compared with normal tissues, the expression levels of KCTD2, KCTD9, KCTD10, KCTD12, KCTD15 and KCTD16 in stage 1, stage 2, stage 3, and stage 4 were significantly reduced. The expression levels of KCTD5 and KCTD21 in stage 1, stage 2, stage 3, and stage 4 were significantly increased (Fig. 3A).

Similarly, the expression levels of KCTD2, KCTD9, KCTD10, KCTD12, KCTD15, and KCTD16 were significantly attenuated in N0, N1, N2, and N3 compared with those in normal tissues. Whereas, the expression levels of KCTD5 and KCTD21 were significantly enhanced in N0, N1, N2, and N3 (Fig. 3B). These results preliminarily suggest that KCTD family genes are related to lymph node metastasis of LUAD.

Finally, we analyzed the correlation between KCTD family genes and TP53 mutations (Fig. 3C). The experimental results demonstrated that the expression levels of KCTD2, KCTD5 and KCTD21 were significantly different between the TP53-Mutant and TP53-Nonmutant groups, suggesting that these genes were related to the mutation status of TP53.

### Prognostic roles of KCTD family genes in patients with LUAD

**Figure 4:**
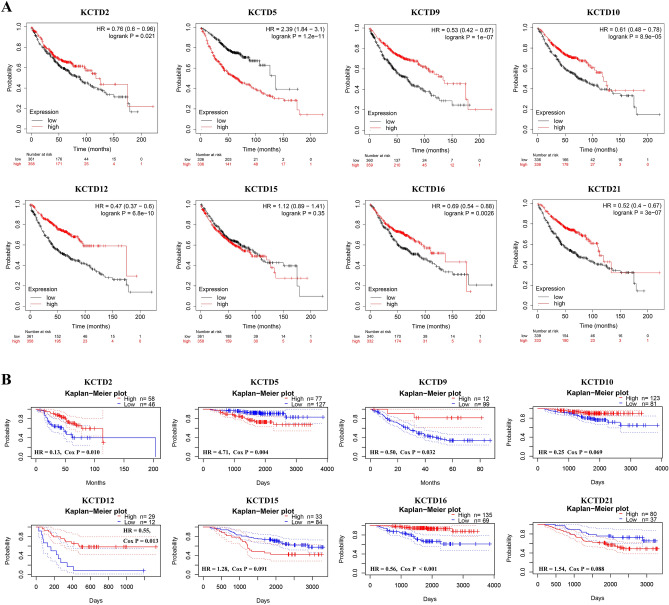
Survival analysis of KCTD family genes in lung cancer in Kaplan–Meier plotter (**A**) and PrognoScan (**B**) databases. Overall survival (OS) was used as an indicator. *P* < 0.05 was considered statistically significant.

The Kaplan–Meier Plotter database was used to identify the prognostic value of KCTD family genes in LUAD (Fig. 4A, Table 1). The OS of the patients was used as an indicator. The results showed that there was a significant difference in OS between the high expression group and the low expression group of KCTD2 (*P* < 0.05), KCTD5 (*P* < 0.001), KCTD9 (*P* < 0.001), KCTD10 (*P* < 0.001), KCTD12 (*P* < 0.001), KCTD16 (*P* < 0.01) and KCTD21 (*P* < 0.001). Among them, the OS of the high expression group of KCTD2, KCTD9, KCTD10, KCTD12 and KCTD16 was longer than that of the low expression group, and the high expression of these genes suggested a better prognosis in LUAD patients. However, the OS of the group with high KCTD5 expression (63.40 months) was significantly shorter than that of the group with low KCTD5 expression (136.33 months). Furthermore, the PrognoScan survival analysis demonstrated that the high and low expression of KCTD2 (HR = 0.13, Cox *P* = 0.010), KCTD5 (HR = 4.71, Cox *P* = 0.004), KCTD9 (HR = 0.50, Cox *P* = 0.032), KCTD12 (HR = 0.55, Cox *P* = 0.013) and KCTD16 (HR = 0.56, Cox *P* < 0.001) were significantly correlated with the OS of lung cancer patients (Fig. 4B). Similarly, the OS of KCTD5 low expression group was significantly longer than that of KCTD5 high expression group (Fig. 4B). These results suggest that KCTD5 may be associated with the poor prognosis of LUAD and can be used as a prognostic marker.

**Table 1:** Survival analysis of KCTD family genes (Kaplan–Meier plotter).

Gene	Samples	Cut-off value	Expression (range of probe)	Median survival		*P*-value	HR (95% CI)
				Low expression cohort (months)	High expression cohort (months)		
KCTD2	719	239	23–791	88.70	117.33	< 0.05	0.76 (0.60–0.96)
KCTD5	672	128	23–690	136.33	63.40	< 0.001	2.39 (1.84–3.10)
KCTD9	719	539	75–2084	69.00	133.57	< 0.001	0.53 (0.42–0.67)
KCTD10	672	535	152–1281	76.00	119.87	< 0.001	0.61 (0.48–0.78)
KCTD12	719	1983	153–12,493	63.40	175.00	< 0.001	0.47 (0.37–0.60)
KCTD15	719	128	5–1196	107.00	92.97	0.35	1.12 (0.89–1.41)
KCTD16	672	25	1–491	89.00	136.33	< 0.01	0.69 (0.54–0.88)
KCTD21	672	222	18–731	71.27	112.67	< 0.001	0.52 ( 0.40–0.67)

### Association of KCTD family genes with immune infiltration level in LUAD

**Figure 5:**
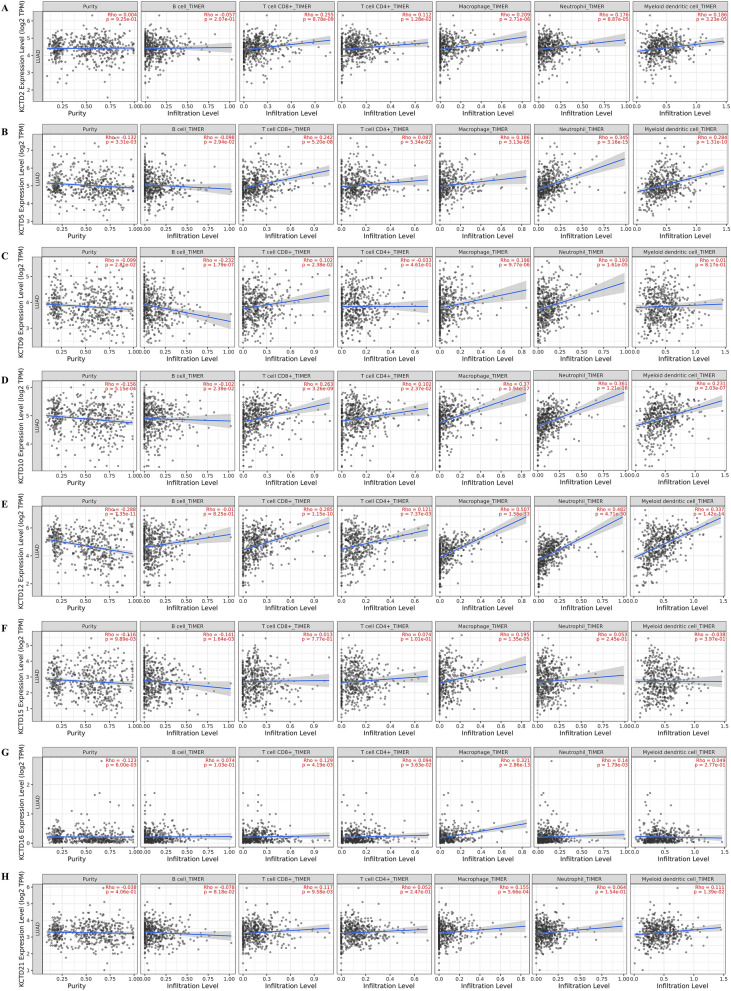
Association of KCTD family genes with immune infiltration level in LUAD (TIMER2.0). (**A**–**H**) Correlations between KCTD family genes (KCTD2, KCTD5, KCTD9, KCTD10, KCTD12, KCTD15, KCTD16 and KCTD21) and tumor infiltrating immune cells in LUAD. The Spearman correlation coefficient was used, absolute value of Rho greater than 0.1 and *P* value less than 0.05 were defined as the correlation between KCTD family genes and immune cells.

We further investigated the correlation of KCTD expression with the immune infiltration levels in LUAD. As shown in Fig. 5, KCTD10 was related to all six immune infiltrates. KCTD2 and KCTD12 were positively correlated with CD4^+^ T cells, CD8^+^ T cells, neutrophils, macrophages and dendritic cells but had no correlation with B cells. All the differentially expressed KCTDs were positively correlated with macrophages. Except for KCTD15, all the other differentially expressed KCTDs were positively correlated with CD8^+^ T cells.

### Genetic alteration and interaction analyses of KCTDs in LUAD

**Figure 6:**
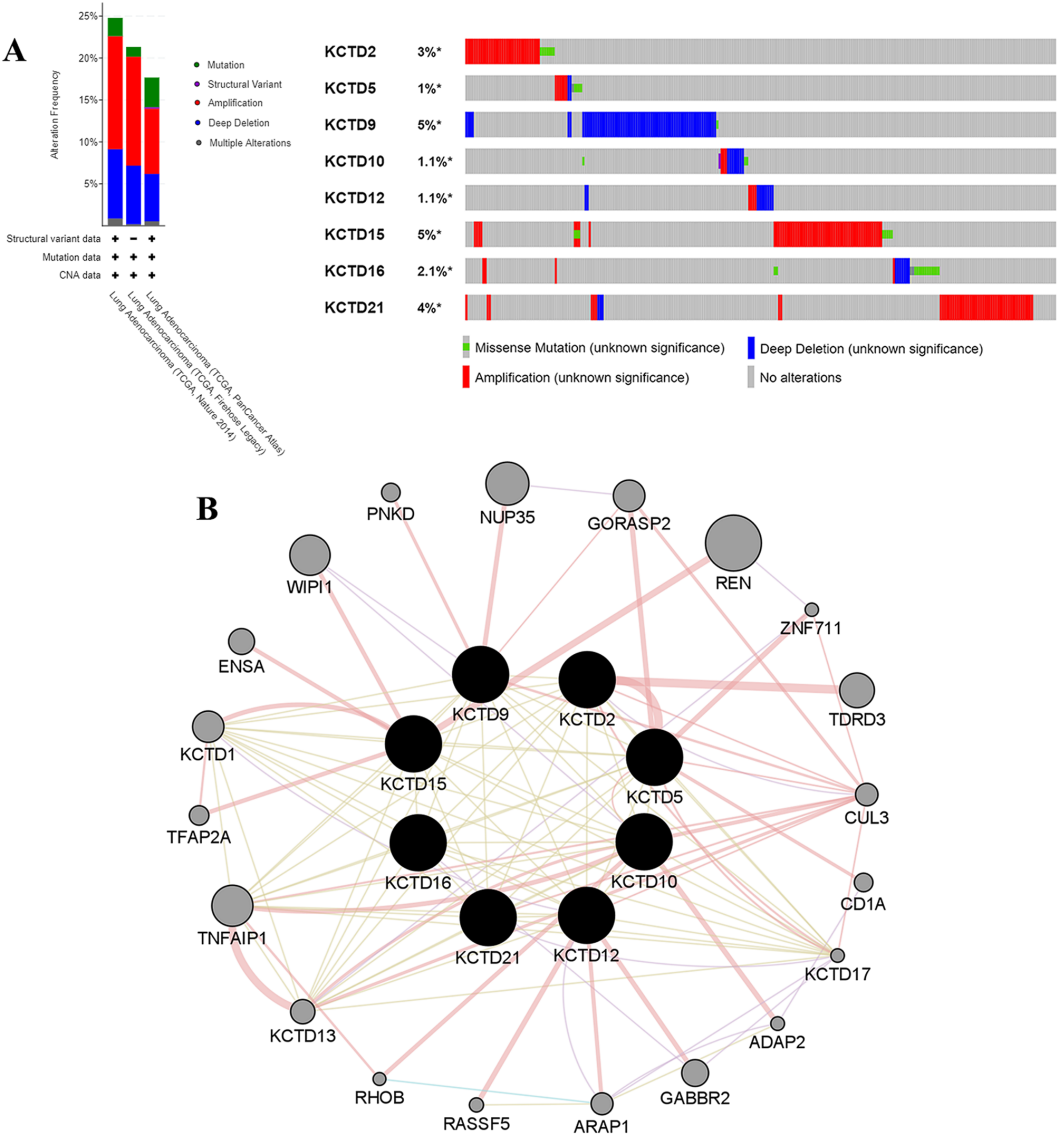
Genetic alteration and interaction analyses of KCTDs in LUAD. (**A**) Genetic alteration of KCTD family genes in LUAD (cBioPortal). (**B**) Analysis of co-expression and interaction genes of KCTD family genes (GeneMANIA).

Subsequently, the genetic alterations of KCTD family members in LUAD were analyzed using the cBioPortal database (Fig. 6A). Among a series of genetic alterations, amplification and deep deletion were the most common. The KCTD family genes with the most to least genetic alterations in LUAD are as follows: KCTD9 (5%), KCTD15 (5%), KCTD21 (4%), KCTD2 (3%), KCTD16 (2.1%), KCTD10 (1.1%), KCTD12 (1.1%), and KCTD5 (1%). Furthermore, GeneMANIA was used to analyze the co-expression and interactions of KCTD family genes (Fig. 6B). The 8 central nodes representing KCTD family genes (KCTD2, KCTD5, KCTD9, KCTD10, KCTD12, KCTD15, KCTD16 and KCTD21) were surrounded by 20 nodes which represent genes closely correlated with the family.

### KCTDs co-expression networks in LUAD

**Figure 7:**
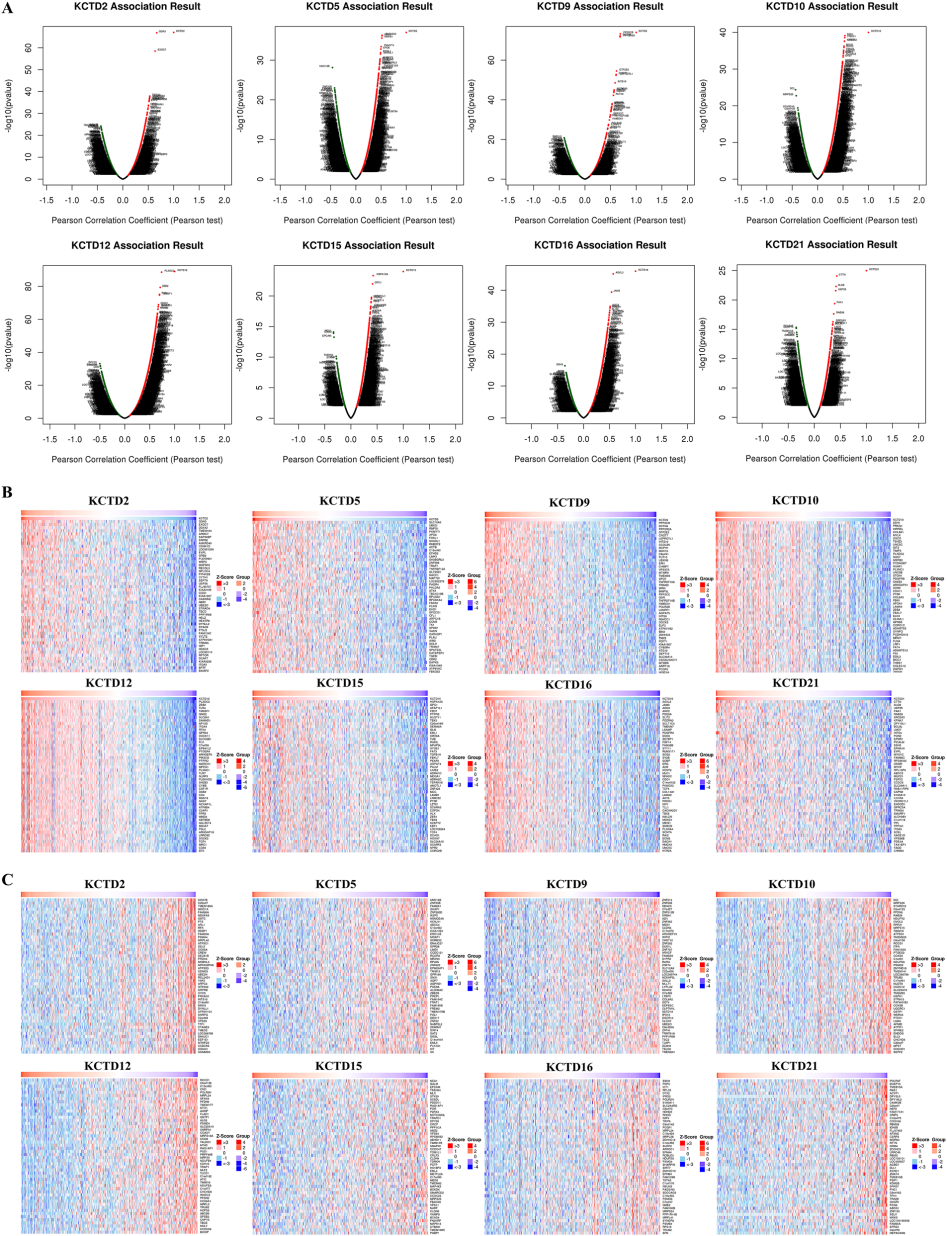
KCTDs co-expression networks in LUAD (LinkedOmics). (**A**) The volcano plot of KCTDs co-expression genes. Red dots represent genes that are positively associated with KCTDs and green dots represent genes that are negatively associated with KCTDs. Heat maps showed the top 50 positively (**B**) and negatively (**C**) co-expressed genes of KCTDs in LUAD.

We explored the KCTDs co-expression networks in LUAD using LinkedOmics. As shown in the volcano plot (Fig. 7A), 5223 genes (red dots) were positively correlated with KCTD2, and 4768 genes (green dots) were negatively associated with KCTD2 (*P* < 0.05); 4846 genes were positively related with KCTD5, and 5622 genes were negatively correlated with KCTD5 (*P* < 0.05); 4077 genes were positively correlated with KCTD9, and 4741 genes were negatively associated with KCTD9 (*P* < 0.05); 6480 genes were positively correlated with KCTD10, and 4545 genes were negatively associated with KCTD10 (*P* < 0.05); 7509 genes were positively related with KCTD12, and 5458 genes were negatively correlated with KCTD12 (*P* < 0.05); 4987 genes were positively correlated with KCTD15, and 2512 genes were negatively associated with KCTD15 (*P* < 0.05); 7047 genes were positively related with KCTD16, and 3479 genes were negatively correlated with KCTD16 (*P* < 0.05); 2749 genes were positively correlated with KCTD21, and 5126 genes were negatively associated with KCTD21 (*P* < 0.05). The top 50 positively (Fig. 7B) or negatively (Fig. 7C) co-expressed genes associated with KCTDs were shown in the heat map.

### Diagnostic features of KCTD family genes in patients with LUAD

**Figure 8:**
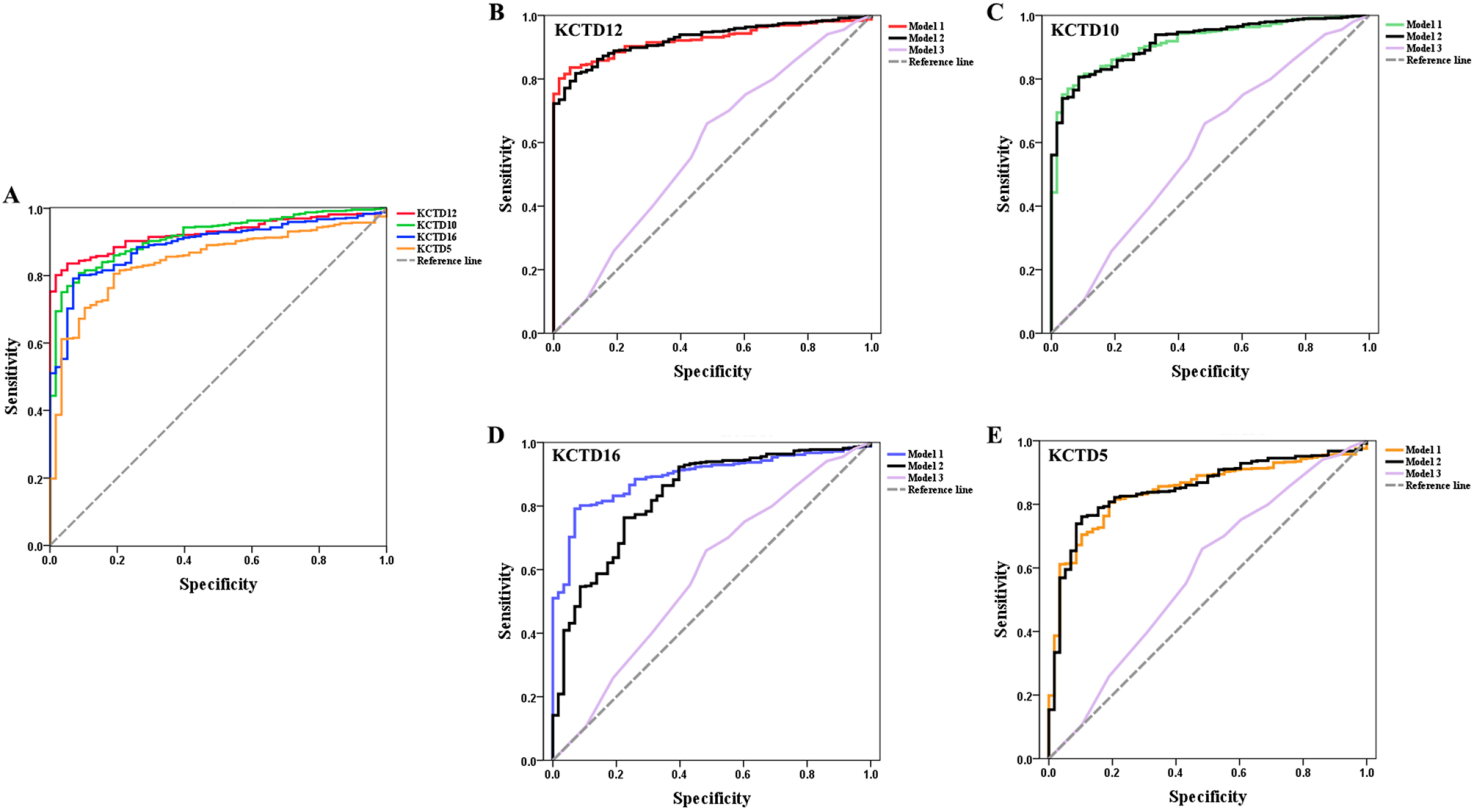
Diagnostic features of KCTD family genes in patients with LUAD. (**A**) ROC analysis showed that KCTDs (KCTD12, KCTD10, KCTD16, KCTD5) can clearly distinguish LUAD from normal samples. Three prediction models, namely Model 1 (including only target genes), Model 2 (including target genes and clinicopathological parameters) and Model 3 (excluding target genes). (**B**) KCTD12. AUC_Model 1_ = 0.924, *P* < 0.001; AUC_Model 2_ = 0.926, *P* < 0.001; AUC_Model 3_ = 0.585, *P* < 0.05. (**C**) KCTD10. AUC_Model 1_ = 0.916, *P* < 0.001; AUC_Model 2_ = 0.916, *P* < 0.001; AUC_Model 3_ = 0.585, *P* < 0.05. (**D**) KCTD16. AUC_Model 1_ = 0.892, *P* < 0.001; AUC_Model 2_ = 0.830, *P* < 0.001; AUC_Model 3_ = 0.585, *P* < 0.05. (**E**) KCTD5. AUC_Model 1_ = 0.845, *P* < 0.001; AUC_Model 2_ = 0.851, *P* < 0.001; AUC_Model 3_ = 0.585, *P* < 0.05. The AUC of Model 1 and Model 2 was close to and significantly larger than Model 3. The red line is KCTD12, the green line is KCTD10, the blue line is KCTD16, and the orange line is KCTD5. The black line is Model 2, the purple line is Model 3.

To identify the diagnostic value of KCTD family genes in LUAD, we performed logistic regression analysis and ROC analysis. As shown in Table 2 and Fig. 8A, KCTD12 (*P* < 0.001, AUC = 0.924), KCTD10 (*P* < 0.001, AUC = 0.916), KCTD16 (*P* < 0.001, AUC = 0.892), KCTD5 (*P* < 0.001, AUC = 0.845), KCTD9 (*P* < 0.001, AUC = 0.760), KCTD21 (*P* < 0.001, AUC = 0.758), KCTD15 (*P* < 0.001, AUC = 0.699), and KCTD2 (*P* < 0.001, AUC = 0.614) could clearly distinguish LUAD from normal samples. Next, we investigated the influence of clinicopathological parameters (age, gender, tumor stage, and smoking history) on the diagnostic model through logistic regression analysis (Table 3).

**Table 2:** ROC analysis of KCTD family genes.

	AUC	*P*-value	95% CI
KCTD12	0.924	< 0.001	0.902–0.947
KCTD10	0.916	< 0.001	0.887–0.946
KCTD16	0.892	< 0.001	0.859–0.925
KCTD5	0.845	< 0.001	0.802–0.888
KCTD9	0.760	< 0.001	0.721–0.799
KCTD21	0.758	< 0.001	0.707–0.809
KCTD15	0.699	< 0.001	0.642–0.757
KCTD2	0.614	< 0.001	0.560–0.669

**Table 3:** Logistic regression analysis: KCTD family genes and clinicopathological characteristics as covariates.

Variable	*P*-value	OR	95% CI
**KCTD2**	0.030	0.464	0.232–0.928
Age (< 65 vs. ≥ 65 )	0.930	0.976	0.561–1.697
Gender (Female vs. Male)	0.895	1.038	0.594–1.813
Clinical stage (I–II vs. III–IV)	0.319	0.724	0.384–1.366
Smoking history (yes vs. no)	0.023	0.525	0.301–0.915
**KCTD5**	< 0.001	13.980	6.648–29.397
Age (< 65 vs. ≥ 65 )	0.458	1.256	0.688–2.295
Gender (Female vs. Male)	0.594	1.178	0.645–2.149
Clinical stage (I–II vs. III–IV)	0.304	0.697	0.350–1.388
Smoking history (yes vs. no)	0.016	0.475	0.259–0.872
**KCTD9**	< 0.001	0.252	0.147–0.431
Age (< 65 vs. ≥ 65 )	0.645	0.874	0.493–1.550
Gender (Female vs. Male)	0.366	1.304	0.733–2.320
Clinical stage (I–II vs. III–IV)	0.516	0.807	0.421–1.544
Smoking history (yes vs. no)	0.009	0.466	0.262–0.830
**KCTD10**	< 0.001	0.002	0.000–0.008
Age (< 65 vs. ≥ 65 )	0.341	1.392	0.705–2.749
Gender (Female vs. Male)	0.421	0.756	0.382–1.495
Clinical stage (I–II vs. III–IV)	0.152	0.556	0.249–1.242
Smoking history (yes vs. no)	0.353	0.717	0.355–1.447
**KCTD12**	< 0.001	0.076	0.041–0.140
Age (< 65 vs. ≥ 65 )	0.834	0.930	0.472–1.834
Gender (Female vs. Male)	0.279	0.686	0.347–1.357
Clinical stage (I–II vs. III–IV)	0.045	0.436	0.193–0.980
Smoking history (yes vs. no)	0.049	0.501	0.252–0.997
**KCTD15**	< 0.001	0.419	0.280–0.628
Age (< 65 vs. ≥ 65 )	0.893	0.962	0.548–1.690
Gender (Female vs. Male)	0.691	1.122	0.637–1.974
Clinical stage (I–II vs. III–IV)	0.649	0.860	0.449–1.649
Smoking history (yes vs. no)	0.023	0.519	0.295–0.915
**KCTD16**	< 0.001	0.019	0.005–0.074
Age (< 65 vs. ≥ 65 )	0.859	0.948	0.526–1.710
Gender (Female vs. Male)	0.451	1.258	0.692–2.285
Clinical stage (I–II vs. III–IV)	0.322	0.712	0.363–1.396
Smoking history (yes vs. no)	0.010	0.462	0.256–0.834
**KCTD21**	< 0.001	11.369	4.848–26.658
Age (< 65 vs. ≥ 65 )	0.774	1.088	0.611–1.936
Gender (Female vs. Male)	0.614	0.161	0.651–2.070
Clinical stage (I–II vs. III–IV)	0.405	0.753	0.387–1.467
Smoking history (yes vs. no)	0.012	0.475	0.256–0.851

To exclude the influence of clinicopathological parameters on the performance of KCTD family genes, we further built three prediction models, namely Model 1 (including only target genes), Model 2 (including target genes and clinicopathological parameters) and Model 3 (excluding target genes) (Table 4, Fig. 8B–E). In this part, we focused on genes with AUCs greater than 0.8 (KCTD12, KCTD10, KCTD16, and KCTD5). The AUCs of Model 1 and Model 2 of the KCTD12 gene were very close (AUC_Model 1_ = 0.924, *P* < 0.001; AUC_Model 2_ = 0.926, *P* < 0.001), both greater than 0.9, while the AUC of Model 3 was less than 0.6 (AUC_Model 3_ = 0.585, *P* < 0.05) (Fig. 8B). Similar results were also found for KCTD10 (Fig. 8C), KCTD16 (Fig. 8D), and KCTD5 (Fig. 8E), and the AUCs of Model 1 and Model 2 were close to and significantly greater than that of Model 3. The results suggested that these four target genes play a decisive role in the prediction model and can be used as independent diagnostic factors.

**Table 4:** ROC analysis and KCTDs diagnostic model.

	AUC	*P*-value	95% CI
**KCTD12**			
Model 1	0.924	< 0.001	0.902–0.947
Model 2	0.926	< 0.001	0.902–0.950
Model 3	0.585	0.035	0.503–0.667
**KCTD10**			
Model 1	0.916	< 0.001	0.887–0.946
Model 2	0.916	< 0.001	0.891–0.947
Model 3	0.585	0.035	0.503–0.667
**KCTD16**			
Model 1	0.892	< 0.001	0.859–0.925
Model 2	0.830	< 0.001	0.775–0.886
Model 3	0.585	0.035	0.503–0.667
**KCTD5**			
Model 1	0.845	< 0.001	0.802–0.888
Model 2	0.851	< 0.001	0.808–0.895
Model 3	0.585	0.035	0.503–0.667

Model 1: The included variable is target gene;

Model 2: The included variables include target gene, Age, Gender, Tumor stage, Smoking history;

Model 3: The included variables include Age, Gender, Tumor stage, Smoking history.

### Potential mechanism of the oncogenic effect of KCTD5 in LUAD

**Figure 9:**
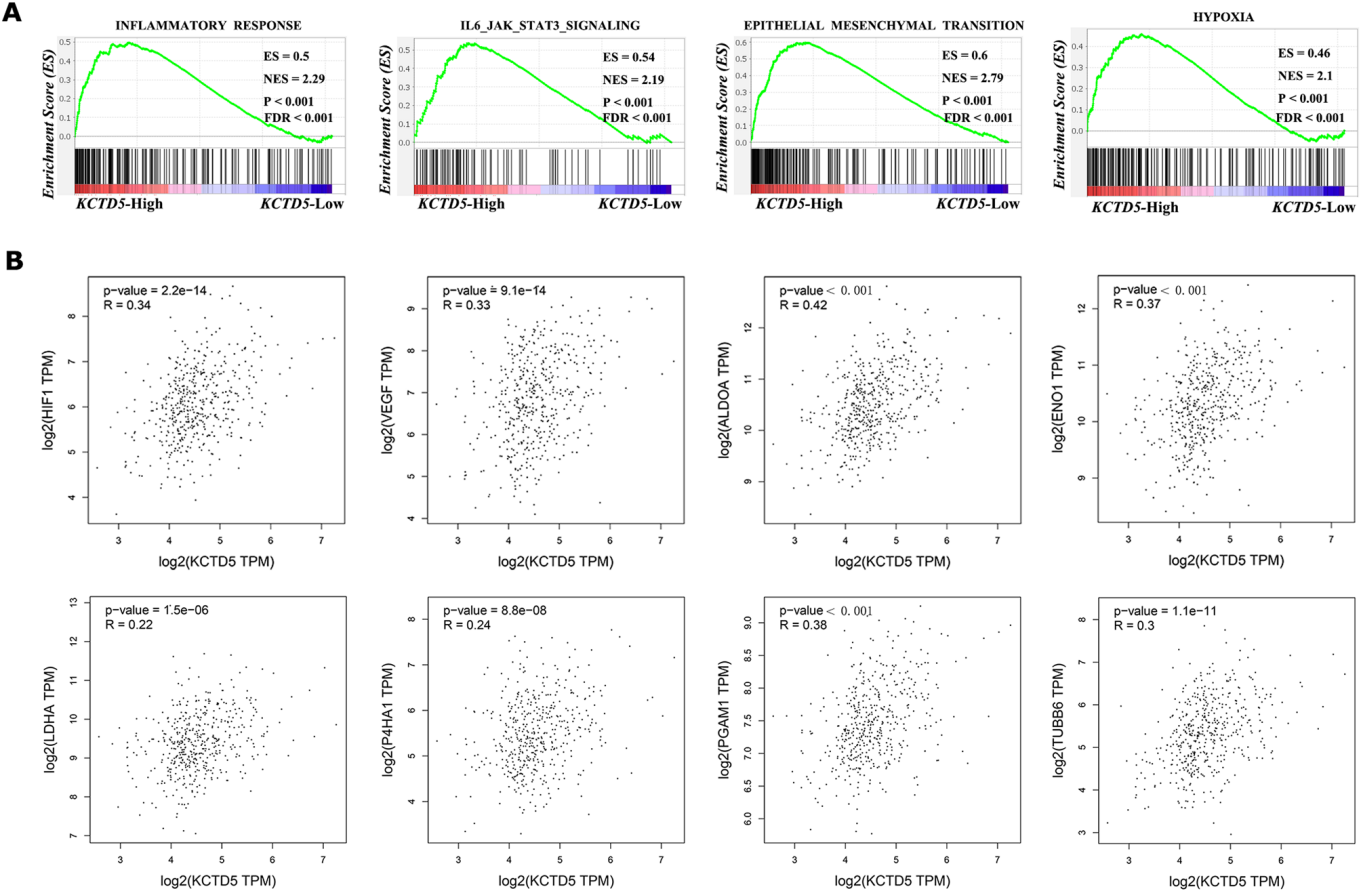
Potential mechanism of the oncogenic effect of KCTD5 in LUAD. (**A**) Gene set enrichment analysis. High expression of KCTD5 enriches signaling pathways associated with malignant progression of tumors, including the inflammatory response, IL6/JAK/STAT3 signaling pathway, EMT and hypoxia. (**B**) Association analysis showed that KCTD5 was positively correlated with hypoxia-related genes such as HIF1, VEGF, ALDOA, ENO1, LDHA, P4HA1, PGAM1 and TUBB6. *P* < 0.05 was considered statistically significant. *ES* enrichment score; *NES* normalized enrichment score; *R* pearson correlation coefficient.

To further explore the potential mechanism of the regulation of KCTD family genes in LUAD, we performed GSEA and correlation analysis. Based on previous studies, we focused on KCTD5, which is highly expressed in LUAD and has bright prospects in prognosis prediction and diagnosis and may be used as a therapeutic target for LUAD. High KCTD5 expression was enriched in many signaling pathways associated with the malignant progression of tumors, including the inflammatory response (*P* < 0.001, FDR < 0.001, NES = 2.29), IL6/JAK/STAT3 signaling pathway (*P* < 0.001, FDR < 0.001, NES = 2.19), epithelial-mesenchymal transition (EMT) (*P* < 0.001, FDR < 0.001, NES = 2.79), and hypoxia (*P* < 0.001, FDR < 0.001, NES = 2.1) (Fig. 9A). It is well known that the hypoxic microenvironment is a crucial factor leading to tumor metastasis. We further tested the association between KCTD5 and hypoxia-related genes, and the results showed that KCTD5 was positively correlated with HIF1 (*P* < 0.001, R = 0.34), VEGF (*P* < 0.001, R = 0.33), ALDOA (*P* < 0.001, R = 0.42), ENO1 (*P* < 0.001, R = 0.37), LDHA (*P* < 0.001, R = 0.22), P4HA1 (*P* < 0.001, R = 0.24), PGAM1 (*P* < 0.001, R = 0.38), and TUBB6 (*P* < 0.001, R = 0.30) (Fig. 9B). These results reminder that KCTD5 may promote the metastasis of LUAD by regulating the hypoxic microenvironment, and the specific molecular mechanism needs to be further studied. KCTD5 may be a new therapeutic target for LUAD.

## Discussion

The study focused on LUAD because it is the predominant subtype of non-small cell lung cancer (NSCLC)^23^. LUAD is a heterogeneous disease with a variety of somatic mutations, including some driver mutations, such as KRAS, EGFR, and ALK^24^. Molecular-targeted therapies against driver gene mutations have dramatically changed the treatment strategies for LUAD^25^. However, the treatment of LUAD also faces great challenges, such as drug resistance and tumor metastasis. Thus, it is necessary to identify novel, stable and reliable molecular markers for early screening and targeted therapy.

Previous studies have shown that KCTD family genes are involved in the regulation of tumorigenesis. KCTD15 inhibits the Hedgehog signaling pathway by increasing the stability of the tumor suppressor KCASH2, thereby inhibiting the proliferation of medulloblastoma cells^26^. Zhong et al. showed that KCTD12 promotes tumorigenesis by activating the CDC25B/CDK1/Aurora A axis-mediated G_2_/M transition^27^. Murakami et al. demonstrated that the CUL3/KCTD10/RhoB axis may serve as a new therapeutic target for HER2-positive breast cancer^28^. Based on early high-throughput microarray screening, we comprehensively explored the expression patterns, diagnostic value, prognostic value, immune infiltration levels, genetic mutation status, and enrichment of downstream signaling pathways of KCTDs in LUAD.

In the present study, the expression of KCTD family genes in LUAD was detected from the transcriptional level and protein expression level, and a series of differentially expressed KCTDs were identified. Surprisingly, we found that the expression of KCTD2 is inconsistent at the transcription level and translation level, the same situation also occurs on KCTD21. As we all know, gene expression is generally regulated by both transcription and translation, and the correlation between mRNA and corresponding protein expression depends on many regulatory factors and metabolic processes. From RNA to protein and then to phenotype is a complex and delicate process. After the formation of mRNA, regulatory activities such as post-transcriptional regulation, epigenetic modification and post-translational regulation are likely to occur, which are important factors leading to the disconformity of mRNA expression trend with its protein level. In addition, background noise from sequencing was also one of the reasons for the low mRNA- protein correlation. Subsequently, we analyzed the correlation between KCTDs gene expression level and clinicopathologic features, and we found that the expression of KCTD5 and KCTD21 was significantly higher in the lymph node metastasis group than in the normal group. This finding suggests that KCTD5 and KCTD21 may be involved in the regulation of tumor metastasis. Importantly, GSEA showed that high KCTD5 expression was positively correlated with EMT. It is well known that EMT plays an important role in tumor metastasis, malignant progression, tumor stemness and anti-apoptosis^29,30^.

Interestingly, we also found that KCTD5 positively regulates tumor hypoxia signaling pathways. Further association analysis showed that KCTD5 was positively correlated with hypoxia-related genes such as hypoxia-inducible factor 1 (HIF1) and vascular endothelial growth factor (VEGF). Hypoxia is a widespread phenomenon in most solid tumors and is closely related to tumor proliferation, differentiation, angiogenesis, energy metabolism and drug resistance^31,32^. At present, hypoxia is becoming recognized as an important driver of EMT in cancer^33,34^. KCTD5 is a member of the KCTD protein family, and its role in lung cancer has not yet been reported. Thus, we propose a new question: does KCTD5 mediate tumor metastasis by regulating hypoxia-induced EMT? In the future, we will further explore the molecular function and regulatory mechanism of KCTD5 in LUAD to provide a new molecular target for the diagnosis and treatment of LUAD, as well as a new mechanism explanation for the metastasis of LUAD.

**Figure 10:**
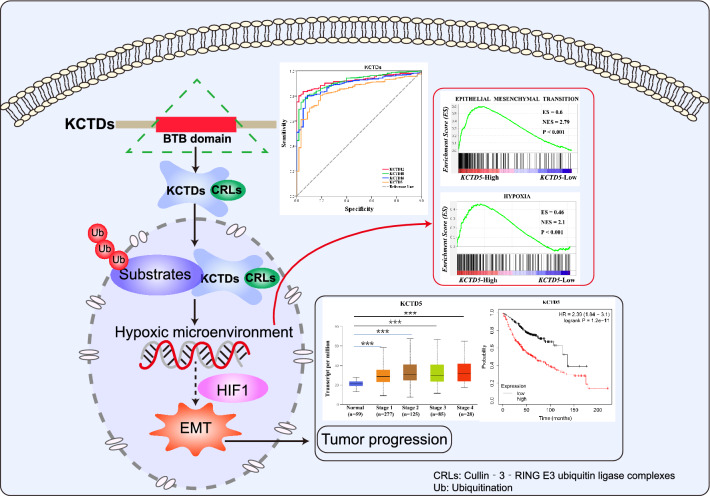
Summary of the potential function and clinical significance of KCTD family genes in LUAD.

In conclusion, our study identified the expression pattern, clinical application value, immune infiltration and molecular function of KCTD family genes in LUAD, and KCTD5 as an important prognostic biomarker may be involved in the regulation of tumor progression mediated by hypoxic microenvironment (Fig. 10). KCTD family genes, especially KCTD5, may serve as new therapeutic targets for LUAD.

## Supplementary Information

Supplementary Information.
