# Supplementary material for: Comprehensive analysis of KCTD family genes associated with hypoxic microenvironment and immune infiltration in lung adenocarcinoma
Source: Sci Rep. 2022 Jun 15;12:9938. doi: 10.1038/s41598-022-14250-6 (PMC9200823; doi:10.1038/s41598-022-14250-6)
Supplement: Supplementary file 1 — Supplementary Information. [file 41598_2022_14250_MOESM1_ESM.docx]

Comprehensive analysis of KCTD family genes associated with hypoxic microenvironment and immune infiltration in lung adenocarcinoma

Yuan-Xiang Shi^1,^ *, Wei-Dong Zhang^2^, Peng-Hui Dai^3^, Jun Deng^4^, Li-Hong Tan^1,^ *

^1^Institute of Clinical Medicine, Hunan Provincial People’s Hospital, The First Affiliated Hospital of Hunan Normal University, Changsha, Hunan 410005, P.R. China;

^2^Respiratory Medicine, Hunan Provincial People’s Hospital, The First Affiliated Hospital of Hunan Normal University, Changsha, Hunan 410005, P.R. China;

^3^Department of Pathology, Hunan Provincial People’s Hospital, The First Affiliated Hospital of Hunan Normal University, Changsha, Hunan 410005, P.R. China;

^4^Department of Pharmacy, Hunan Provincial People’s Hospital, The First Affiliated Hospital of Hunan Normal University, Changsha, Hunan 410005, P.R. China.

Correspondence to: Dr. Yuan-Xiang Shi, Institute of Clinical Medicine, Hunan Provincial People’s Hospital, The First Affiliated Hospital of Hunan Normal University, Changsha, Hunan 410005, P.R. China. E-mail: [yuanxiangshi@hunnu.edu.cn](mailto:yuanxiangshi@hunnu.edu.cn)

Correspondence to: Prof. Li-Hong Tan, Institute of Clinical Medicine, Hunan Provincial People’s Hospital, The First Affiliated Hospital of Hunan Normal University, Changsha, Hunan 410005, P.R. China. E-mail address: Tanlihong118@163.com

**Supplementary materials**


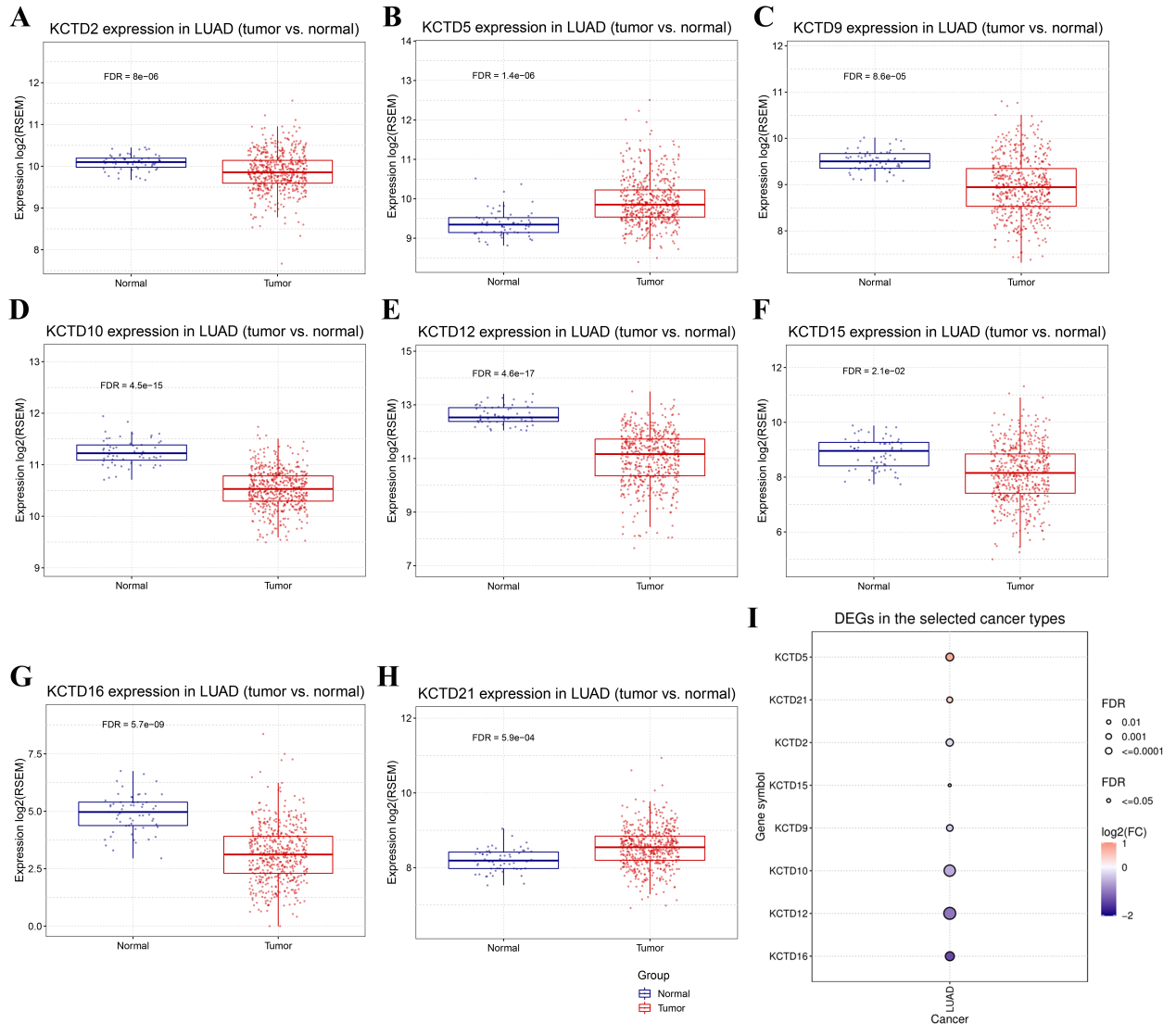


Supplementary Figure 1 The mRNA expression of KCTD family genes in LUAD and normal lung tissues (GSCA database). (A-H) The mRNA expression of KCTD2, KCTD9, KCTD10, KCTD12, KCTD15 and KCTD16 in LUAD was significantly decreased, while KCTD5 and KCTD21 was significantly increased. Figure I summarizes the differentially expressed KCTD family genes between tumor and normal in LUAD. FDR<0.05 was considered statistically significant.
